# Vertical Orientation of Liquid Crystal on Comb-Like 4-(*trans*-4-alkylcyclohexyl)phenoxymethyl-substituted Polystyrene Containing Liquid Crystal Precursor

**DOI:** 10.3390/polym13091404

**Published:** 2021-04-26

**Authors:** Kyutae Seo, Hyo Kang

**Affiliations:** BK-21 Four Graduate Program, Department of Chemical Engineering, Dong-A University, 37 Nakdong-Daero 550 Beon-gil, Saha-gu, Busan 604-714, Korea; kyutae@donga.ac.kr

**Keywords:** anisotropic material, liquid crystal, orientation layer, polystyrene, 4-(*trans*-4-alkylcyclohexyl)phenol

## Abstract

We synthesized a series of polystyrene derivatives modified with precursors of liquid crystal (LC) molecules, including 4-(*trans*-4-ethylcyclohexyl)phenol (homopolymer PECH and copolymer PECH#; # = 5, 10, 15, 20, 40, 60, and 80, where # indicates the molar fraction of 4-(*trans*-4-ethylcyclohexyl)phenoxymethyl in the side chain), 4-(*trans*-4-propylcyclohexyl)phenol (PPCH), 4-(*trans*-4-butylcyclohexyl)phenol (PBCH), and 4-(*trans*-4-amylcyclohexyl)phenol (PAmCH) via polymer modification reactions in order to investigate the orientation of LC molecules on polymer films exhibiting part of the LC molecular structure. A stable and uniform vertical orientation of LC molecules was observed in LC cells fabricated with PECH#, having 15 mol% or more of 4-(*trans*-4-ethylcyclohexyl)phenoxymethyl side groups. In addition, the vertical orientation of LC molecules was observed in LC cells fabricated with homopolymers of PECH, PPCH, PBCH, and PAmCH. The water contact angle on the polymer films could be associated with the vertical orientation of the LC molecules in the LC cells fabricated with polymer films. For example, a vertical LC orientation was observed when the water contact angle of the polymer films was higher than ~81°. Good orientation stability was observed at 200 °C and 15 mW/cm^2^ of UV irradiation for LC cells fabricated with PECH films.

## 1. Introduction

Liquid crystals (LCs) are anisotropic materials which have not only an intermediate phase in which they demonstrate fluidity, but also exceptional physicochemical characteristics due to the positional and/or orientational order of their molecules [1,2,3,4,5]. LCs can be categorized into two specific types according to their physical parameters and environments in order to exhibit liquid crystalline properties: lyotropic and thermotropic LCs. Lyotropic LCs consisting of amphiphiles tend to form self-assembled structures according to their concentration in solution. Their unique structures, including lamellar, hexagonal, and cubic phases, as well as the physicochemical properties of lyotropic LC systems, make them suitable for pharmaceutical applications as drug delivery carriers with various potential applications [6,7,8,9,10]. For example, the cubic structure of lyotropic LC molecules has been investigated extensively as a technology for drug delivery systems because of the great flexibility of the lyotropic LC structure [11,12,13,14]. Therefore, it is possible to upload active ingredients into pharmaceutical drugs with a wide range of polarities and sizes by adjusting the concentration of lyotropic LC systems. Thermotropic LCs exhibit various liquid crystalline phases as a function of temperature. Thermotropic LCs with rod-like shapes can be subdivided broadly into two main classes, nematic and smectic, according to the extent of positional order. Nematic LCs have a characteristic long-range orientational order, which is indicated by the unit vector *n* (called the director). In addition, the anisotropic physicochemical properties of nematic LC molecules, such as refractive index and dielectric tensor, can be applied in electronic devices, including in displays and sensors, as nematic LC molecules are susceptible to external electric and/or magnetic fields. The uniform orientation of thermotropic LC molecules such as nematic LCs has been used for these applications. For example, the vertical orientation of nematic LCs, wherein the director of the nematic LCs is oriented vertically to the surface of the substrate, has been studied for sensor applications because of its susceptibility to small perturbations and binding events [15,16,17,18], which can be observed by an optical apparatus such as a polarized optical microscopy (POM) [19,20]. It has been demonstrated that LC molecules can be aligned by the anisotropic characteristics of a surface via numerous contact and noncontact methods, such as mechanical rubbing, stretching, lithography, polarized ultraviolet (UV) radiation, and ion beam treatment [21,22,23,24,25,26,27,28]. Among these, the mechanical rubbing of polymeric surfaces is the most commonly used contact method to obtain a uniform orientation of LC molecules because of its simplicity and rapidity [29,30]. Films of polyimide derivatives have been widely employed as LC orientation layers using the rubbing technique as the polymer films provide considerable stability in the LC orientation [31,32]. However, hard baking processes are needed to produce polyimide orientation layers, and the conventional hard baking temperature of polyimide films is generally over 200 °C, which is too high for the manufacture of flexible plastic products [33,34]. In addition, unexpected problems have been detected after the rubbing process, such as dust generation, physical damage, and electrostatic charge on the orientation layer surfaces [35,36,37]. Noncontact methods for the orientation of LC molecules have been investigated to overcome the drawbacks of the rubbing method. Photoalignment has been proposed as a promising noncontact orientation technology for next-generation LC display applications, such as flexible displays, because of the advantages of photoalignment, such as cleanliness, lack of restrictions with respect to surface morphology, and suitability for large glass substrates. Numerous polymers, including a variety of photoreactive functional groups for photoisomerization, photodimerization, and photodegradation have been studied as photoalignment layers [38,39,40]. The orientation of LC molecules on polymer films is dependent on the chemical composition of the polymer films [41]. Polystyrene (PS) surfaces producing planar LC orientation can be prepared at low temperatures suitable for the fabrication of flexible displays. However, it is generally accepted that the anchoring stability of LC cells fabricated with PS films is not good enough to produce reliable LC orientation layers; the planar LC orientation of these cells cannot be sustained for more than one day. Recently, vertical LC orientation layers using PS derivatives via a polymer substitution reaction have been developed for electro-optical applications, including flexible displays, due to their advantages such as low temperature processability and superior optical transparency. PS derivatives, grafted with natural extracts, long alkyl groups, or fluoroalkyl groups, have been developed in order to orient LC molecules vertically on substrates using noncontact methods. For example, the vertical orientation of LC molecules in LC cells fabricated with PS derivatives substituted with natural extracts such as capsaicin, eugenol, and vanillin, was observed when the substituent ratio was larger than 60 mol%. This is due to the long alkyl groups of the natural extracts, which are related to low surface energy owing to the steric effect of alkyl groups on the polymer film surface [42,43,44,45,46,47,48]. The surface energy of polymer films and the molecular orientation of the polymers are decisive factors in obtaining vertical LC orientation behavior due to steric repulsion and/or interactions between LC molecules and surfaces [49,50].

**Figure 1:**
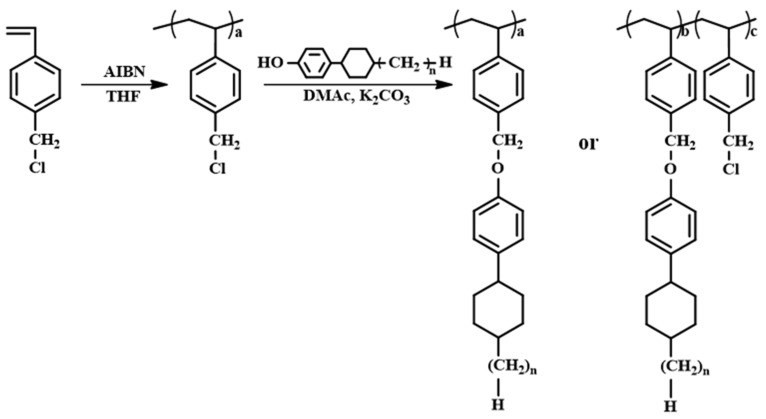
Synthetic route to 4-(*trans*-4-ethylcyclohexyl)phenoxymethyl (PECH# and PECH, *n* = 2), 4-(*trans*-4-propylcyclohexyl)phenoxymethyl (PPCH, *n* = 3), 4-(*trans*-4-butylcyclohexyl)phenoxymethyl (PBCH, *n* = 4), and 4-(*trans*-4-amylcyclohexyl)phenoxymethyl-substituted polystyrene (PAmCH, *n* = 5), where # represents the molar fraction of 4-(*trans*-4-ethylcyclohexyl)phenol containing monomeric units in the polymer.

In this study, we synthesized a series of PS derivatives with 4-(*trans*-4-alkylcyclohexyl)phenoxymethyl side groups (Figure 1) in order to systematically investigate LC behaviors on the orientation layer fabricated with PS derivatives structurally similar to LC molecules. The vertical orientation of LC molecules in the LC cells fabricated with these polymer films was observed when the substituent ratio was only 15 mol%. These results suggest that the similarity between the chemical structure of the orientation layer and LC molecules can be advantageous in the vertical orientation of LC molecules. The synthesis and characterization of these polymers and the optical properties of the assembled LC cells with unrubbed polymer films were studied.

## 2. Materials and Methods

### 2.1. Materials

4-Chloromethylstyrene, 4′-pentyl-4-biphenylcarbonitrile (5CB, *n_e_* = 1.7360, *n_o_* = 1.5442, and Δ*ε* = 14.5, where *n_e_*, *n_o_*, and Δ*ε* represent the extraordinary refractive index, ordinary refractive index, and dielectric anisotropy, respectively), and silica gel were purchased from Merck Co. (Seoul, Korea). Benzophenone, sodium, and hexane were purchased from Aldrich Co. (Seoul, Korea). 4-(*Trans*-4-ethylcyclohexyl)phenol, 4-(*trans*-4-propylcyclohexyl)phenol, 4-(*trans*-4-butylcyclohexyl)phenol, and 4-(*trans*-4-amylcyclohexyl)phenol were obtained from Tokyo Chemical Industry Co. (Tokyo, Japan). Potassium carbonate, 2,2′-azobisisobutyronitrile (AIBN), tetrahydrofuran (THF), molecular sieve (4 Å), and *N*,*N*′-dimethylacetamide (DMAc) were acquired from Daejung Chemicals & Metals Co. (Siheung, Korea). Methanol was supplied by SK Chemical Co. (Ulsan, Korea). DMAc and ethanol were dried over molecular sieves. THF was dried by refluxing with benzophenone and sodium, followed by distillation. 4-Chloromethylstyrene was purified by column chromatography on silica gel using hexane as an eluent to remove any impurities and inhibitors (*tert*-butylcatechol and nitroparaffin). AIBN was purified by crystallization using methanol. Poly(4-chloromethylstyrene) (PCMS) was synthesized through conventional free radical polymerization of 4-chloromethylstyrene using 2,2′-azobisisobutyronitrile (AIBN) under a nitrogen atmosphere. The mixture in solution was cooled to room temperature and poured into methanol to obtain a white precipitate. The precipitate was further purified by Soxhlet extraction using hot methanol to remove the remaining monomer (4-chloromethylstyrene) and low molecular weight PCMS. AIBN was used as the initiator. All other reagents and solvents were used as received.

^1^H NMR of PCMS (400 MHz, CDCl_3_, *δ*/ppm): *δ* = 1.01–1.88 (–*CH_2_*–*CH*–Ph–, 3H), *δ* = 4.13–4.77 (–Ph–*CH_2_*–Cl, 2H), *δ* = 6.00–7.22 (CH_2_–CH–*PhH*–CH_2_–, 4H).

### 2.2. Preparation of 4-(trans-4-alkylcyclohexyl)phenoxymethyl Modified Polystyrene

The following procedure was used to synthesize 4-(*trans*-4-alkylcyclohexyl)phenoxymethyl-substituted polystyrenes (PACH), in which the alkyl group is –(CH_2_)_n_H (*n* = 2, 3, 4, and 5). The synthesis of 4-(*trans*-4-ethylcyclohexyl)phenoxymethyl-substituted polystyrene (PECH) is given as an example. A mixture of PCMS (0.300 g, 1.97 mmol), 4-(*trans*-4-ethylcyclohexyl)phenol (0.605 g, 2.96 mmol, and 150 mol% compared with PCMS), and potassium carbonate (0.491 g, 3.55 mmol, 120 mol% compared with 4-(*trans*-4-ethylcyclohexyl)phenol) in DMAc (50 mL) was heated to 70 °C with magnetic stirring at 200 rpm under a nitrogen atmosphere for 24 h. Thereafter, the mixture in solution was cooled to room temperature and then poured into methanol to obtain a white precipitate. The precipitate was further purified by several reprecipitations from DMAc solution into methanol, and then a Soxhlet extractor was used to remove potassium carbonate and the remaining salts with hot methanol. A yield of 80% PECH was obtained after drying overnight under vacuum.

^1^H NMR of PECH (400 MHz, CDCl_3_, *δ*/ppm): *δ* = 0.51–1.50 (–*CH_2_*–CH–Ph–, –*(CH_2_)_2_–CH–CH_2_–CH_3_*, 12H), *δ* = 1.70–2.54 (–CH_2_–*CH*–Ph–, –O–Ph–*CH–(CH_2_)_2_*–, 6H), *δ* = 4.62–5.07 (–Ph–*CH_2_*–O–, 2H), *δ* = 6.12–7.22 (–CH_2_–CH–*PhH*–CH_2_–, –O–*PhH*–Cy–, 8H).

Similarly, 4-(*trans*-4-propylcyclohexyl)phenoxymethyl (PPCH, *n* = 3), 4-(*trans*-4-butylcyclohexyl)phenoxymethyl (PBCH, *n* = 4), and 4-(*trans*-4-amylcyclohexyl)phenoxymethyl-substituted polystyrene (PAmCH, *n* = 5) were synthesized using the aforementioned procedure. Here, 4-(*trans*-4-propylcyclohexyl)phenol (0.646 g, 2.96 mmol, 150 mol% compared with PCMS), 4-(*trans*-4-butylcyclohexyl)phenol (0.688 g, 2.96 mmol, 150 mol% compared with PCMS), and 4-(*trans*-4-amylcyclohexyl)phenol (0.729 g, 2.96 mmol, 150 mol% compared with PCMS), respectively, replaced 4-(*trans*-4-ethylcyclohexyl)phenol.

^1^H NMR of PPCH (400 MHz, CDCl_3_, *δ*/ppm): *δ* = 0.61–1.48 (–*CH_2_*–CH–Ph–, –*(CH_2_)_2_–CH–CH_2_–CH_2_–CH_3_*, 14H), *δ* = 1.64–2.64 (–CH_2_–*CH*–Ph–, –O–Ph–*CH–(CH_2_)_2_*–, 6H), *δ* = 4.52–5.18 (–Ph–*CH_2_*–O–, 2H), *δ* = 6.13–7.19 (–CH_2_–CH–*PhH*–CH_2_–, –O–*PhH*–Cy–, 8H).

^1^H NMR of PBCH (400 MHz, CDCl_3_, *δ*/ppm): *δ* = 0.69–1.51 (–*CH_2_*–CH–Ph–, –*(CH_2_)_2_–CH–CH_2_–CH_2_–CH_2_–CH_3_*, 16H), *δ* = 1.66–2.50 (–CH_2_–*CH*–Ph–, –O–Ph–*CH–(CH_2_)_2_*–, 6H), *δ* = 4.60–5.05 (–Ph–*CH_2_*–O–, 2H), *δ* = 6.15–7.22 (–CH_2_–CH–*PhH*–CH_2_–, –O–*PhH*–Cy–, 8H).

^1^H NMR of PAmCH (400 MHz, CDCl_3_, *δ*/ppm): *δ* = 0.62–1.49 (–*CH_2_*–CH–Ph–, –*(CH_2_)_2_–CH–CH_2_–CH_2_–CH_2_–CH_2_–CH_3_*, 18H), *δ* = 1.64–2.55 (–CH_2_–*CH*–Ph–, –O–Ph–*CH–(CH_2_)_2_*–, 6H), *δ* = 4.54–5.00 (–Ph–*CH_2_*–O–, 2H), *δ* = 6.16–7.22 (–CH_2_–CH–*PhH*–CH_2_–, –O–*PhH*–Cy–, 8H).

Copolymers of PECH, designated as PECH#, where # is the degree (mol%) of substitution of chloromethyl to the 4-(*trans*-4-ethylcyclohexyl)phenoxymethyl group, were prepared using the same procedure as PECH. For example, PECH5, PECH10, PECH15, PECH20, PECH40, PECH60, and PECH80 were prepared using 0.020 g (0.10 mmol), 0.040 g (0.20 mmol), 0.060 g (0.29 mmol), 0.080 g (0.39 mmol), 0.161 g (0.79 mmol), 0.241 g (1.18 mmol), and 0.322 g (1.58 mmol) of 4-(*trans*-4-ethylcyclohexyl)phenol, respectively, using a slight excess of potassium carbonate (120 mol% compared with 4-(*trans*-4-ethylcyclohexyl)phenol).

### 2.3. Film Preparation and LC Cell Assembly

Solutions of PECH#, PACH (PECH, PPCH, PBCH, and PAmCH) in THF (1 wt.%) were filtered using a poly(tetrafluoroethylene) (PTFE) membrane with a pore size of 0.45 μm. Then, thin polymer films were prepared by spin-coating (2000 rpm, 60 s) onto 2.0 × 2.5 cm^2^ glass substrates. LC cells were fabricated by assembling two polymeric layers onto two glass substrates using spacers with a thickness of 4.25 μm. The physicochemical properties of 4-pentyl-4-cyanobiphenyl (5CB), such as surface tension, have been documented in numerous studies due to its accessible nematic temperature range near room temperature, high positive dielectric anisotropy, and remarkable chemical stability. Therefore, 5CB was selected to fabricate LC cells to investigate the correlation between the orientation layer and LC molecules via physicochemical interactions [51,52,53,54]. The cells were filled with the nematic LC (5CB). The manufactured LC cells were then sealed using epoxy glue.

### 2.4. Instrumentation

^1^H-nuclear magnetic resonance (NMR) spectroscopy using an MR400 DD2 (Agilent Technologies, Inc., Santa Clara, CA, USA) NMR spectrometer, differential scanning calorimetry (DSC) using a Q-10 (TA Instruments, Inc., New Castle, DE, USA), and polarized optical microscopy (POM) images of LC cells using a Nikon Eclipse E600 POL (NIKON, Inc., Tokyo, Japan) equipped with a polarizer and Nikon Coolpix 995 digital camera (NIKON, Inc., Tokyo, Japan) were employed for the characterization of the synthesized materials. The static contact angles of water on the polymer films were determined using a Kruss DSA10 (KRÜSS Scientific Instruments Inc., Hamburg, Germany) contact angle analyzer equipped with drop shape analysis software (KRÜSS Scientific Instruments Inc., Hamburg, Germany). The contact angles for each sample were measured more than four times on three independently prepared films, and the average values were used. The ultraviolet (UV) stability test of the LC cells was conducted using a VL-6.LC lamp (λ_max_ = 365 nm, Vilber Lourmat, Paris, France) with intensities of 5, 10, and 15 mW/cm^2^ during the 30 min in order to corroborate the reliability to apply severe environment. The exposure dose of irradiated UV light on the LC cells was measured with a UV detector using GT-513 (Giltron, Seoul, Korea).

## 3. Results and Discussion

**Figure 2:**
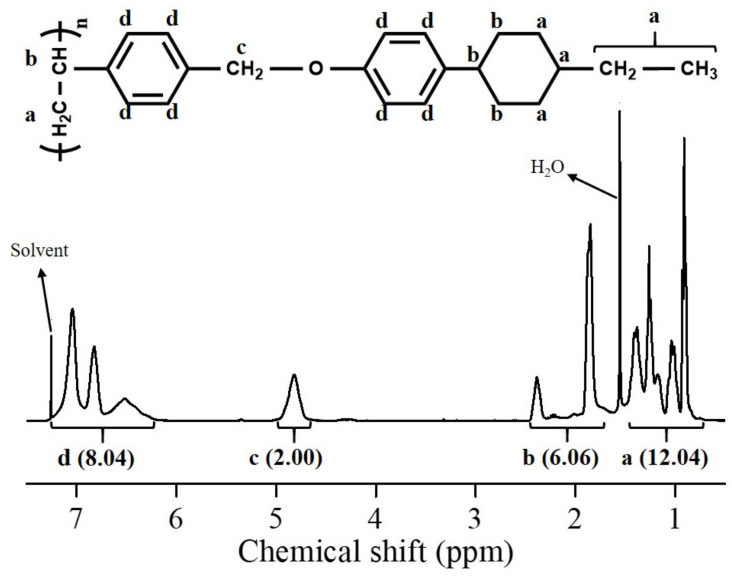
^1^H nuclear magnetic resonance (NMR) spectrum of PECH.

The synthetic routes to the PACH homopolymers (PECH, PPCH, PBCH, and PAmCH) and copolymers PECH# (PECH5, PECH10, PECH15, PECH20, PECH40, PECH60, and PECH80) are shown in Figure 1. Copolymers with different substitution ratios (mol%) were obtained by varying the molar ratio of 4-(*trans*-4-ethylcyclohexyl)phenol in the reaction mixture. Approximately 100% conversion of chloromethyl to oxymethyl was obtained when an excess (150 mol%) of the phenols was reacted with poly(4-chloromethylstyrene) at 70 °C for 24 h. Figure 2 shows the ^1^H NMR spectrum of PECH as an example. The chemical composition of the monomeric units in the obtained polymers was confirmed using ^1^H NMR spectroscopy. The ^1^H NMR spectrum and assignment of the respective peaks of PECH are shown in Figure 2. The ^1^H NMR spectrum of PECH indicates the presence of protons from PECH derivatives (*δ*/ppm = 0.51–1.50 (12H), 1.70–2.54 (6H), 4.62–5.07 (2H), 6.12–7.22 (8H); peaks a, b, c, and d. The degree of substitution from chloromethyl to oxymethyl was calculated to be approximately 100% by comparing the integrated area of the oxymethyl peak in the range 4.62–5.07 ppm and the phenyl group peaks in the range 6.12–7.22 ppm. Similar integrations and calculations for PECH# and PACH were performed and were typically within ±10% of the expected values. These polymers were soluble in medium-polarity solvents with low boiling points, such as tetrahydrofuran (THF) and chloroform, and in aprotic polar solvents, including *N*,*N*′-dimethylformamide (DMF) and *N,N′*-dimethylacetamide (DMAc). The solubility of the polymer samples in various solvents was sufficient for PECH# and PACH to be applied as thin-film materials. Among the organic solvents, THF was chosen as the coating solvent for thin-film fabrication because of its low eco-toxicity and good biodegradability [55]. In addition, these polymer thin-films can be fabricated at a low temperature based on a wet process, owing to their good solubility in volatile organic solvents.

**Figure 3:**
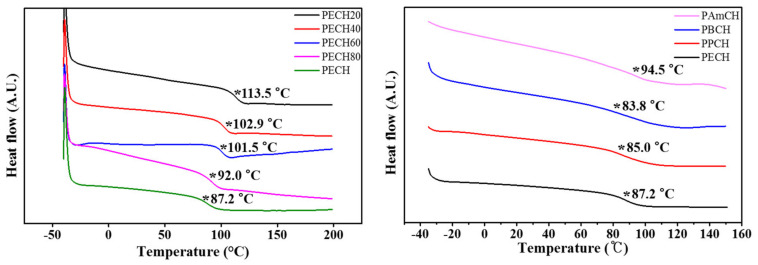
Differential scanning calorimetry (DSC) thermogram of PECH# (PECH20, PECH40, PECH60, and PECH80) and PACH (PECH, PPCH, PBCH, and PAmCH) (* indicates the glass transition).

The thermal properties of the polymers were studied using differential scanning calorimetry (DSC) at a heating and cooling rate of 10 °C/min under a nitrogen atmosphere. All polymers were amorphous, and only one glass transition was observed in the DSC thermograms. The glass transition temperatures were determined from the extrapolated intersection of the asymptotes to the glassy and rubbery regions for enthalpy [56,57], as illustrated in Figure 3. As the molar content of the 4-(*trans*-4-ethylcyclohexyl)phenoxymethyl side group increased from 20 mol% to 100 mol%, the *T_g_* value decreased from 113.5 °C for PECH20 to 87.2 °C for PECH. In addition, as the number of carbon atoms in the alkyl moiety of the 4-(*trans*-4-alkylcyclohexyl)phenoxymethyl side group increased from 2 to 4, the *T_g_* decreased from 87.2 °C for PECH to 83.8 °C for PBCH. The decrease in *T_g_* values of the polystyrene derivatives with increasing bulkiness of the side groups was previously reported and ascribed to an increase in free volume of the polymer, as polymers having larger free volumes have lower *T_g_* values [55,56,57]. However, as the number of carbon atoms in the side group increased from 4 to 5, the *T_g_* increased from 83.8 °C for PBCH to 94.5 °C for PAmCH. The increase in the polystyrene derivative *T_g_* values was ascribed to an increase in the number of carbon atoms in the side groups, due to π–π and van der Waals interactions [58,59,60,61,62].

**Figure 4:**
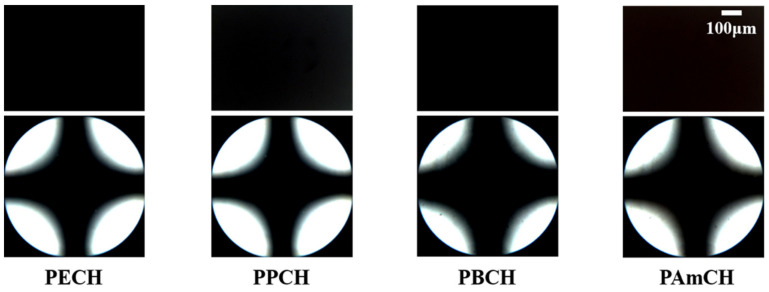
Orthoscopic (top) and conoscopic (bottom) polarized optical microscopy (POM) images of the LC cells fabricated with PECH, PPCH, PBCH, and PAmCH films.

It is known that the molecular orientation of LCs can be affected by the chemical structure of the orientation layer, owing to interactions at the interface between the LC molecules and the orientation layer [63,64,65]. Therefore, LC cells made from films of polystyrene derivatives substituted with precursors of LC molecules, such as 4-(*trans*-4-ethylcyclohexyl)phenoxymethyl, 4-(*trans*-4-propylcyclohexyl)phenoxymethyl, 4-(*trans*-4-butylcyclohexyl)phenoxymethyl, and 4-(*trans*-4-amylcyclohexyl)phenoxymethyl in the side chain, were fabricated using 5CB in order to investigate the orientation behavior of the LC molecules on polymer films with LC-like moieties. The vertical LC orientation of LC cells fabricated with PECH, PPCH, PBCH, and PAmCH films can be observed by the Maltese cross pattern in the conoscopic POM images, as shown in Figure 4. In addition, LC cells fabricated from PECH, PPCH, PBCH, and PAmCH films were stable over several months.

**Figure 5:**
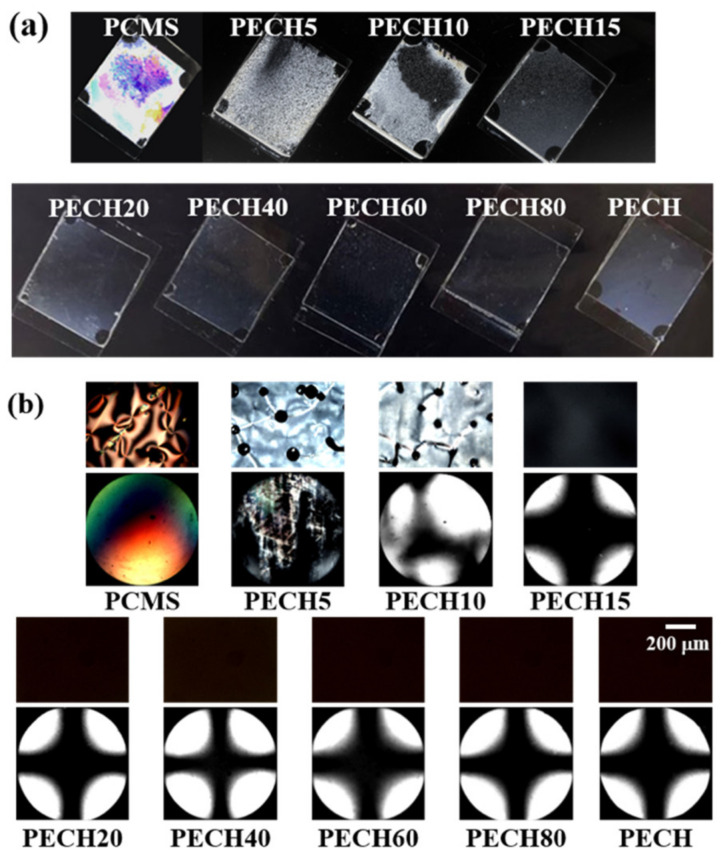
(**a**) Photograph; (**b**) orthoscopic (top) and conoscopic (bottom) polarized optical microscopy (POM) images of the LC cells fabricated with PCMS, PECH# (PECH5, PECH10, PECH15, PECH20, PECH40, PECH60, and PECH80), and PECH.

We also investigated the effect of the amount of 4-(*trans*-4-ethylcyclohexyl)phenoxymethyl moieties in the side chain on the vertical LC orientation properties. Figure 5a shows photographs of the LC cells made from the PCMS, PECH#, and PECH films. A random planar LC orientation was observed for the LC cell fabricated with the PCMS and PECH5 films. On the other hand, a partial vertical orientation of the LC molecules was observed in a small area of the LC cell fabricated with the PECH10 film. The LC cells fabricated with the PECH# films having 15 mol% or more of the 4-(*trans*-4-ethylcyclohexyl)phenoxymethyl side group, including PECH15, PECH20, PECH40, PECH60, PECH80, and PECH, showed a uniform vertical LC orientation behavior in the entire photographed area of the images of the LC cells. Moreover, the LC orientation behavior of LC cells fabricated with the PECH# and PECH films was investigated by observing their POM images for a more accurate analysis (Figure 5b). A random planar or a partial vertical LC orientation was observed in the LC cells fabricated using the PCMS, PECH5, and PECH10 films. However, the PECH#, having 15 mol% or more of 4-(*trans*-4-ethylcyclohexyl)phenoxymethyl side groups (PECH15, PECH20, PECH40, PECH60, and PECH80), and PECH films were able to produce a stable vertical LC orientation, as shown by their POM images, including dark orthoscopic images and the Maltese cross patterns of conoscopic images.

No discernible differences in the LC orientation of PECH15, PECH20, PECH40, PECH60, PECH80, and PECH films, according to the molar fraction of the 4-(*trans*-4-ethylcyclohexyl)phenoxymethyl in the side groups, could be observed in the Maltese cross patterns of the conoscopic POM images. The vertical orientation of the LC molecules in the cell fabricated with PECH15 could be observed due to the LC-like chemical structure of the side chains in the polymer. Therefore, we believe that the similarity of the molecular structure between the orientation layer and LC molecules could be advantageous for the vertical orientation of LC molecules in devices.

**Figure 6:**
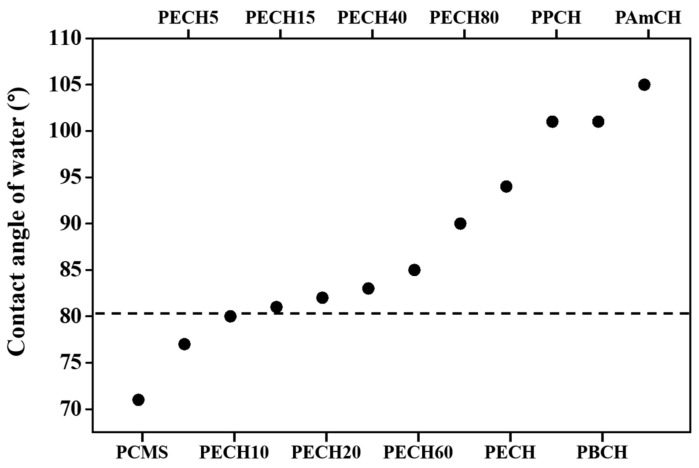
Water contact angle values and LC behaviors of polymer films. The upper part and lower part around the dashed line indicate vertical and planar LC orientation behaviors, respectively.

It is important to understand the relationship between the orientation behavior of the LC molecules and the surface properties of the polymer films in several areas of LC applications. It is known that LC molecules can be oriented vertically on the surface when the surface energy of the substrate is smaller than the surface tension of the LC [66,67,68]. It is well known that the low surface energy of polymer films, hydrophobic character of the surface, and low wettability has been implicated in the high contact angles of water on the polymer films [69,70]. Therefore, the static contact angle of water on the PECH# and PACH films was measured to investigate the effect of wettability on the LC orientation (Table 1 and Figure 6). The vertical orientation of LCs on PECH15, PECH20, PECH40, PECH60, PECH80, PECH, PPCH, PBCH, and PAmCH films was observed. The contact angles of water on the polymer films were 81, 82, 83, 85, 90, 94, 101, 101, and 105°, respectively. However, the LC cells fabricated with PCMS, PECH5, and PECH10 which had water contact angles of 71, 77, and 80°, respectively, did not show uniform vertical LC orientation behavior. Therefore, the vertical LC orientation behavior could be ascribed to the hydrophobic character of the polymer film, such as the water contact angle of approximately 81°.

**Figure 7:**
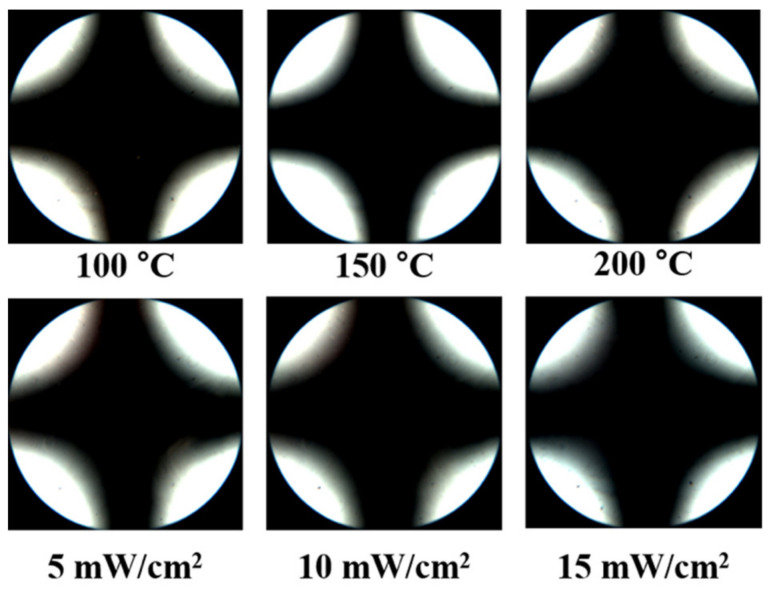
Conoscopic polarized optical microscopy (POM) images of the LC cells made using PECH films, after thermal treatment at 100, 150, and 200 °C for 10 min and UV treatment having intensities of 5, 10, and 15 mW/cm^2^ for 30 min, respectively.

It was considered appropriate to use chemically inert homopolymers (PACH) in order to fabricate LC devices because of the chemical reactivity of the chloromethyl group in the copolymer (PECH#). Among the series of prepared polymers, PECH was chosen as a promising material in order to orient the LC molecules, as it showed not only stable bulk thermal properties, confirmed by high its *T_g_* value, but also satisfactory hydrophobicity to fabricate LC cells by the capillary method. Therefore, the reliability evaluation of the LC cells fabricated with PECH was conducted under harsh conditions, such as high temperatures and ultraviolet (UV) irradiation, in order to substantiate LC orientation stability. Figure 7 shows the thermal stability behavior of the LC cells fabricated using PECH films estimated from conoscopic POM images after heating for 10 min at temperatures of 100, 150, and 200 °C. No distinguishable difference in the orientation of the LC molecules on the PECH films can be observed by the Maltese cross pattern in the conoscopic POM images, indicating that the LC cells fabricated with PECH films can be applied to high-temperature conditions. In addition, the UV stability of LC cells made from the PECH films was estimated from conoscopic POM images. Conoscopic POM images of the LC cells were monitored after UV irradiation (λ_max_ = 365 nm) having intensities of 5, 10, and 15 mW/cm^2^ during 30 min. As shown in Figure 7, no discernible differences in the vertical LC orientation on PECH films were observed in the conoscopic POM images, indicating that the vertical LC alignment ability of the LC cells was maintained even at high UV irradiation intensity.

## 4. Conclusions

A copolymer series of 4-(*trans*-4-ethylcyclohexyl)phenoxymethyl-substituted polystyrenes (PECH#) and homopolymer 4-(*trans*-4-alkylcyclohexyl)phenoxymethyl-substituted polystyrenes (PECH, PPCH, PBCH, and PAmCH) was synthesized to evaluate the LC orientation behavior of the polymer films. The vertical LC orientation behavior was observed for the LC cells made from polymers with a higher molar content of 4-(*trans*-4-ethylcyclohexyl)phenoxymethyl side groups. For example, LC cells fabricated with polymers having 15 mol% or more of 4-(*trans*-4-ethylcyclohexyl)phenoxymethyl (PECH15, PECH20, PECH40, PECH60, PECH80, and PECH) side groups exhibited vertical LC orientation, while LC cells fabricated with PCMS, PECH5, and PECH10 films having less than 15 mol% of 4-(*trans*-4-ethylcyclohexyl)phenoxymethyl side group partially showed LC textures with birefringence. The vertical orientation of the LC molecules in the LC cells fabricated with polymer films was observed, despite the short side chain length of PECH and the low substitution ratio of approximately 15 mol%. These results suggest that the similarity between the chemical structure of the orientation layer and LC molecules can be advantageous in the vertical orientation of LC molecules. The vertical LC orientation behavior was well correlated with the polymer films having a water contact angle larger than approximately 81°, owing to the unique structure of the 4-(*trans*-4-alkylcyclohexyl)phenoxymethyl side chain. Therefore, we believe that 4-(*trans*-4-alkylcyclohexyl)phenoxymethyl-substituted polystyrenes can be a potential candidate as an LC orientation layer for next-generation applications with low temperatures based on wet processes.

## Figures and Tables

**Table 1:** Water contact angles and LC orientation behaviors of the polymer films.

Polymer Designation	Water Contact Angle (°) *^a^*	Vertical LC Aligning Ability *^b^*
PCMS	71	X
PECH5	77	X
PECH10	80	∆
PECH15	81	O
PECH20	82	O
PECH40	83	O
PECH60	85	O
PECH80	90	O
PECH	94	O
PPCH	101	O
PBCH	101	O
PAmCH	105	O

*^a^* Measured from static contact angles. *^b^* X: Planar LC orientation; ∆: Partial vertical orientation; O: Uniform vertical LC orientation.

## Data Availability

The data presented in this study are available on request from the corresponding author.
